# The Expression of Alpha-Fetoprotein in Human Blastocoel Fluid-Conditioned Media In Vitro: A Proof of Concept Study

**DOI:** 10.3390/ijms26041722

**Published:** 2025-02-18

**Authors:** Shahryar K. Kavoussi, Shu-Hung Chen, John David Wininger, Arnav Lal, William E. Roudebush, Parviz K. Kavoussi, Amy S. Esqueda, Justin Chen, Renee J. Chosed

**Affiliations:** 1 Austin Fertility & Reproductive Medicine/Westlake IVF, 300 Beardsley Lane, Bldg B, Suite 200, Austin, TX 78746, USA; 2 Department of Biomedical Sciences, School of Medicine Greenville, University of South Carolina, Greenville, SC 29605, USAchosed@greenvillemed.sc.edu (R.J.C.)

**Keywords:** blastocoel fluid, blastocyst, alpha-fetoprotein, AFP, in vitro fertilization, IVF

## Abstract

Alpha-fetoprotein (AFP) is measured during pregnancy in maternal serum to screen for, and in amniotic fluid to test for, neural tube defects. This study aimed to determine whether or not AFP is expressed in blastocoel fluid-conditioned media (BFCM) at the blastocyst stage of embryonic development. For this in vitro study, BFCM was obtained from blastocyst stage embryos following standard embryology laboratory processes. Good quality blastocysts (n = 40) had trophectoderm biopsy for preimplantation genetic testing for aneuploidy (PGT-A) with subsequent blastocyst vitrification and BFCM collection. BFCM samples (n = 40) were analyzed for human AFP protein via an AFP Human ELISA Kit. Statistical analysis was performed with Fisher’s exact test. AFP was expressed in 12.5% (5/40) of BFCM samples (range = 1.69–20.5 pg/mL). Of blastocysts with AFP in BFCM, 80% (4/5) had aneuploid PGT-A results; of blastocysts with no AFP in BFCM, 57% (20/35) had aneuploid PGT-A results, with no difference between groups (*p* = 0.63). Our study demonstrates AFP expression in BFCM. To our knowledge, this is the first study to report the detection of AFP at the embryonic blastocyst stage in vitro. Future studies are needed and underway to determine whether assessment of AFP at the embryonic stage can improve embryo transfer outcomes.

## 1. Introduction

Alpha-fetoprotein (AFP), discovered in 1956 [1], is a 70-kilodalton glycoprotein produced by the human fetus, specifically in the yolk sac and later in the fetal liver. AFP has garnered much attention in the clinical setting of pregnancy, as maternal serum AFP (msAFP) is used in order to screen for neural tube defects (NTDs) and amniotic AFP (aAFP) is used for diagnostic testing of such defects; additionally, first or second trimester ultrasound can detect spina bifida in cases of elevated AFP related to NTDs. Although its role in fetal development is not entirely clear, potential roles in fetal growth and immune system regulation have been proposed [2,3]. One function of AFP is maintenance of oncotic pressure in the fetal intravascular compartment [4]. In addition, it has been suggested that AFP modulates embryonic brain development, based on findings of primary cell culture of rat brain neurons in both in vitro and an ex vivo models [5].

Previous studies have examined AFP in the realm of assisted reproductive technologies (ARTs). Although there have been some conflicting data regarding msAFP in in vitro fertilization (IVF) pregnancies [6,7,8,9,10], the authors of a systematic review concluded that, due to variability in levels reported as well as the lack of ability to conduct a meta-analysis, differences between prenatal screening for msAFP in IVF pregnancies could not be generalized [11]. A prior publication showed that, at the level of first trimester trophoblast cells, semi-quantitative immunohistochemical analysis of aborted pregnancies demonstrated significantly higher AFP expression in the trophoblastic cells in the IVF embryo transfer study group than in the naturally conceived control group. Increased AFP expression in the study group was not associated with differences in obstetric outcomes in comparison to the control group [12]. In addition, AFP has been shown to have higher expression in placentae of pregnancies conceived via IVF when compared with placentae of natural pregnancies [13].

A retrospective study evaluating the potential correlation between embryo morphology, antepartum biomarker level and obstetric outcomes after single embryo transfer (SET) included 78 women with such data. Pregnancies that had resulted from the SET of an embryo with an inner cell mass (ICM) of lower grade showed higher second trimester msAFP levels and AFP multiples of the mean (MoM). There were no differences in obstetric outcomes between lower or higher ICM morphologic grading [14].

Blastocoel fluid (BF) has been shown to be a potential source of noninvasive testing at the blastocyst stage of embryo development. In efforts to search for less invasive methods to karyotype preimplantation embryos than trophectoderm biopsy of blastocyst stage embryos, previous publications have demonstrated the presence of cell-free DNA (cfDNA) in BF. Additionally, correlation with embryonic morphology has been shown [15]. Furthermore, cfDNA content in human blastocoel fluid-conditioned media (BFCM) may provide additional information regarding embryo quality due to the potential to differentiate the euploid versus aneuploid status of embryos [16]. To our knowledge, there have been no previous reported cases demonstrating the presence of AFP at the embryonic blastocyst stage in vitro. This study was conducted to determine whether or not AFP is expressed in BFCM.

## 2. Results

AFP was expressed in 12.5% (5/40) of BFCM samples, ranging from 1.69 pg/mL to 20.5 pg/mL, with levels from each of these five samples shown in Table 1. Of blastocysts with AFP expression in BFCM, 80% (4/5) had aneuploid PGT-A results; of blastocysts with no AFP expression in BFCM, 57% (20/35) had aneuploid PGT-A results. Fisher’s exact test showed no difference in aneuploidy rates between groups, with an odds ratio of 3.095% (confidence interval: 0.03–29.66; *p* value = 0.63). For each of the 40 BFCM samples, data were tabulated for the AFP protein level as measured by ELISA, whether the BFCM sample was from a Day 5 or 6 blastocyst, as well as for corresponding blastocyst grading and the result of PGT-A testing (Table 1). In terms of a potential relationship between blastocyst grading and the euploidy rate, there was no difference in euploidy rates between blastocysts with ICM grade A, 40% (14/35), and ICM grade B, 40% (2/5) (Fisher’s exact test statistic value = 1, *p* < 0.5), and there was no difference in euploidy rates between blastocysts with TE grade A, 35% (8/23), or TE grade B, 47% (8/17) (Fisher’s exact test statistical value = 0.52, *p* < 0.5).

## 3. Discussion

NTDs represent some of the most common serious birth defects, arising when the neural tube of an embryo does not close as it normally would during the first 28 days post-conception. Examples of NTDs include spina bifida and anencephaly. With a reported prevalence of 7 per 10,000 births in the United States, clinical manifestations of NTDs vary from a spectrum of no impairment of function to the more often encountered some degree of sequelae (such as paralysis, hydrocephaly, and urinary incontinence) to lethality [17]. As NTDs can have significant health implications from a morbidity and mortality standpoint, a non-invasive manner to evaluate an embryo with lesser risk of developing NTDs and/or other potential adverse obstetric outcomes, such as spontaneous abortion, would be desired, in addition to euploidy, to select an optimal embryo for transfer to the uterus.

To our knowledge, this is the first publication to report the expression of AFP in BFCM. This proof of concept study demonstrates the expression of AFP in BFCM from five individual blastocysts. Although the presence of AFP in BFCM seemed to be at a higher rate in the aneuploid blastocyst group than in the euploid blastocyst group, there was no statistical difference between these groups, potentially due to small sample sizes. In contrast with a previous study, which suggested that lower ICM grading is associated with higher second trimester msAFP levels [14], each of the five blastocysts in our study that had AFP detected in their BFCM samples had a higher ICM grade of A; however, four of these five blastocysts had trophectoderm biopsies indicating aneuploid status. Interestingly, a prior publication indicated the strong expression of AFP by villous trophoblastic cells in a group with anembryonic pregnancies when compared with a group with viable pregnancies [18]. Furthermore, a recent rapid test strip that detects both AFP and IGFBP-1 has been shown to detect the presence of embryonic or fetal tissue in vaginal blood, thereby aiding in the diagnosis of miscarriage and potentially ruling out ectopic pregnancy [19].

It is possible that AFP levels are secreted by pluripotent cells of a greater proportion of embryos that are at early stages, only several days past Days 5 and 6 of embryonic development, for example, and that the 5 of 40 BFCM samples (12.5%) in this study with detectable AFP levels represent the very earliest threshold for measuring AFP. Moreover, although an association between trophectoderm biopsy and measurable AFP in BFCM is possible, Sigler et al. found that msAFP concentrations at 15–18 weeks of gestation were not different among 417 patients that had chorionic villus sampling 3–10 weeks earlier and 967 patients without chorionic villus sampling [20].

As AFP was less often present in BFCM in this study, studies with larger sample sizes are needed to further evaluate the expression of AFP by pluripotent cells of preimplantation blastocyst stage embryos. Eventually, as embryonic culture medium systems that routinely sustain embryonic development past Day 7 are developed and applied in embryology laboratories for clinical IVF, future studies may provide more information as to whether or not AFP measurement in embryonic cells, BF, and/or embryo-conditioned culture medium has the potential to be clinically useful in order to select euploid embryos that carry less risk of developing into pregnancies with NTDs and/or other adverse obstetric outcomes such as spontaneous miscarriage.

## 4. Materials and Methods

### 4.1. Controlled Ovarian Stimulation, Oocyte Retrieval and Embryology

BFCM samples were obtained during IVF processes as subsequently described. Female patients underwent their planned routine IVF clinical cases, which consisted of controlled ovarian stimulation with subcutaneously administered exogenous gonadotropins as well as subcutaneous gonadotropin hormone antagonist to suppress luteinizing hormone prior to triggering final oocyte maturation with leuprolide acetate and/or recombinant human chorionic gonadotropin (hCG) 35 h prior to transvaginal ultrasound-guided oocyte retrieval. After oocyte isolation and intracytoplasmic sperm injection to achieve IVF, embryos were cultured to Day 5 and Day 6 with the goal of blastocyst formation. Good quality blastocysts were those graded as 2BB or higher, based on Gardner and Schoolcraft’s blastocyst grading system [21]. All 40 of the blastocysts from which BFCM samples were obtained (40/40; 100%) had trophectoderm biopsies for preimplantation genetic testing for aneuploidy (PGT-A) prior to blastocyst vitrification and BFCM collection.

### 4.2. Blastocoel Fluid-Conditioned Medium Sample Collection

As per routine protocol in the embryology laboratory, each blastocyst was placed in an individual medium (Multipurpose Handling Medium containing 10% Synthetic Serum Substitute, Irvine Scientific, Santa Ana, CA, USA) drop for laser-assisted blastocyst collapsing and trophectoderm biopsy. After biopsy, a pipette was used to mix and collect 20 µL of medium into a 0.2 mL PCR tube for storage at −20 °C. Biopsied trophectoderm cells were shipped to a reference genetics laboratory for PGT-A via next generation sequencing.

### 4.3. Enzyme-Linked Immunosorbent Assay for Human AFP Detection

BFCM samples (n = 40) were assessed for the presence of human AFP protein using the AFP Human ELISA Kit (ThermoFisher Cat #EHAFP, Life Technologies Corporation, Carlsbad, CA, USA), which has a sensitivity of 6 pg/mL. Briefly, samples were brought to a total volume of 100 µL with the addition of Assay Diluent B provided with the kit, and then added to the AFP antibody-coated wells in a 96-well plate. Following a 2.5 h incubation at room temperature, the assay was completed according to the manufacturer’s instructions. The absorbance of each well in the assay plate was read using a Tecan Infinite M1000 plate reader at 450 nm and 550 nm. A standard curve was generated to determine the amount of AFP in each blastocoel fluid sample.

### 4.4. Data Analysis

Statistical analysis of nonparametric data was performed via Fisher’s exact test due to small sample sizes. IRB exemption was granted by St. David’s Institutional Review Board because of the de-identified nature of collected data and because BFCM would routinely be discarded in the given IVF clinical cases.

## 5. Conclusions

We demonstrated AFP expression in BFCM samples of preimplantation blastocysts in vitro. Although embryonic culture medium systems past Day 7 with very large sample sizes may be necessary for potential preimplantation data collection with regard to NTDs risk, research to investigate a potential association of AFP expression at the embryonic level and spontaneous miscarriage risk can be studied more efficiently. Such studies are necessary to further assess whether AFP expression at the level of a human blastocyst stage embryo is predictive of maternal and fetal obstetric outcomes of trophectoderm-tested euploid blastocyst transfer.

## Figures and Tables

**Table 1:** Blastocoel fluid-conditioned media (BFCM) samples, alpha-fetoprotein (AFP) protein levels as measured by enzyme-linked immunosorbent assay (ELISA) with Day 5 or 6 of blastocyst formation, as well as corresponding blastocyst grades and results of preimplantation genetic testing for aneuploidy (PGT-A).

BFCM Samples	AFP (pg/mL)	Day 5 or 6	Embryo Grade	PGT-A Result
1	1.692308	5	3AB	Aneuploid
2	8.307692	5	2AA	Aneuploid
3	10.46154	5	2AA	Aneuploid
4	0	5	3BB	Aneuploid
5	0	5	4AA	Euploid
6	0	5	3AA	Euploid
7	0	5	3AA	Aneuploid
8	0	5	3AB	Aneuploid
9	0	5	3AB	Euploid
10	0	5	3BB	Euploid
11	0	5	2AA	Euploid
12	0	5	2AB	Euploid
13	0	5	2BB	Aneuploid
14	0	6	3AB	Aneuploid
15	0	5	4AB	Euploid
16	0	6	2AB	Euploid
17	0	6	3AA	Aneuploid
18	0	6	3AB	Aneuploid
19	0	5	3AA	Euploid
20	0	5	3AA	Euploid
21	0	5	3AA	Euploid
22	20.53846	5	3AA	Euploid
23	0	5	2AA	Aneuploid
24	0	6	2AB	Aneuploid
25	0	5	3AA	Aneuploid
26	0	5	3AA	Aneuploid
27	0	5	3AA	Aneuploid
28	0	5	3AA	Aneuploid
29	0	5	2AA	Aneuploid
30	0	5	2AB	Euploid
31	0	6	2BB	Euploid
32	0	6	3AA	Aneuploid
33	0	6	2AA	Aneuploid
34	0	6	3AB	Aneuploid
35	0	6	3AB	Euploid
36	0	6	2BB	Aneuploid
37	0	5	2AA	Aneuploid
38	1.923078	5	4AA	Aneuploid
39	0	5	3AA	Aneuploid
40	0	5	3AA	Euploid

## Data Availability

Data are contained within this article.
